# Supplementary material for: MetaRibo-Seq measures translation in microbiomes
Source: Nat Commun. 2020 Jun 29;11:3268. doi: 10.1038/s41467-020-17081-z (PMC7324362; doi:10.1038/s41467-020-17081-z)
Supplement: Supplementary file 10 — Supplementary Data 7 [file 41467_2020_17081_MOESM10_ESM.zip › File2/Confidence_VeryHigh_Taxonomy/301816_out.krona.html]

Javascript must be enabled to view this page.

members
magnitude
magnitudeUnassigned
count
unassigned
taxon
rank

301816\_out


SRS104400\_contig\_number\_18324
1
6

superkingdom
2
5

phylum
1239
5

class
5
186801

186802
5
order

186803
5
family

841
5
genus

species

SRS075716\_contig\_number\_contig-100\_1495.1496SRS148721\_contig\_number\_contig-100\_21575.173058
1897018
2

species

SRS014948\_contig\_number\_contig-100\_4715.4716SRS015431\_contig\_number\_contig-100\_3745.257831SRS043411\_contig\_number\_contig-100\_11281.11281
3
1262943
